# Case Report: Comparison different response after neoadjuvant chemotherapy of the breast with cell-in-cell—report of two cases

**DOI:** 10.3389/fmed.2025.1631621

**Published:** 2026-01-20

**Authors:** Danhong Zhan, Zhikang Li, Kaiting Yu, Chenxi Wang, Qiang Sun, Ruizhi Wang, Meifang He

**Affiliations:** 1 Laboratory of General Surgery, The First Affiliated Hospital, Sun Yat-sen University, Guangzhou, China; 2 The Provincial Key Laboratory of Biotechnology, South China University of Technology, Guangzhou, China; 3 Academy of Military Medical Sciences; Research Unit of Cell Death Mechanism, Chinese Academy of Medical Science, Beijing, China; 4 Department of Laboratory Medicine, The First Affiliated Hospital, Sun Yat-sen University, Guangzhou, China

**Keywords:** cell-in-cell structures, neoadjuvant chemotherapy, HER2, variability, case report

## Abstract

Neoadjuvant chemotherapy (NACT) is commonly applied in human epidermal growth factor receptor 2 (HER2)-positive breast cancer to reduce tumor size and increase the likelihood of breast-conserving surgery. However, predictive markers of response remain limited. We report two cases of HER2-positive breast cancer with divergent responses to NACT, highlighting the dynamics of cell-in-cell structures (CICs). Both patients initially presented with palpable breast masses and biopsy-confirmed HER2-positive invasive carcinoma with frequent CICs. After NACT, one patient achieved a favorable pathological response, accompanied by a reduction in CICs frequency. In contrast, the other patient showed limited response, with persistent complex CICs, which led to an adjustment of the adjuvant regimen. These observations suggest that dynamic cell-in-cell (CIC) patterns may reflect heterogeneous tumor responses to NACT, providing insights for individualized treatment planning.

## Introduction

Presently, 15–20% of invasive breast carcinomas overexpress the proto-oncogene human epidermal growth factor receptor 2 (HER2, ERBB2), correlating with an aggressive phenotype and poor prognosis (1). HER2 overexpression results in a poorer prognosis, as it overly activates mitogenic and survival pathways, including the phosphatidylinositol 3-kinase–AKT (PI3K-AKT) and tumor protein p53 (p53) pathways, leading to a decrease in apoptosis and an extension of cell survival (2, 3). HER2 is thus a well-established therapeutic target, and several anti-HER2 drugs such as trastuzumab as a single agent or in conjunction with standard chemotherapy in neoadjuvant chemotherapy (NACT) can substantially enhance the pathological complete response (pCR) rate with significantly better clinical prognosis in patients with HER2-positive breast cancer (4, 5). Numerous clinical trials have shown that NACT can enhance the percentage of patients suitable for breast-conserving surgery, as well as fluctuation of biomarkers such as estrogen receptors (ER) and progesterone receptors (PR) between pre- and post-treatment (6–8). Despite these advances, 40–50% of patients with HER2-positive breast cancer exhibit residual disease after NACT, placing them at higher risk for recurrence and progression (9, 10). This heterogeneity in treatment response highlights the urgent need to explore novel biomarkers and biological phenomena that may underlie or predict treatment efficacy.

The cell-in-cell structure (CICs) is a distinctive and regulated biological phenomenon in which one or more viable cells (referred to as target cells) are internalized into another cell (the host cell), resulting in diverse cellular outcomes with significant implications in tumor biology. Once thought to be a rare artifact, cell-in-cell formation is now recognized as a structured process involved in cancer progression, immune evasion, and therapeutic resistance. Several distinct types of CIC events have been described, including entosis, cannibalism, emperipolesis, and emperitosis, each governed by unique molecular mechanisms and associated with different biological functions (11–15). CICs are broadly categorized into homotypic CICs, which occur between tumor cells of the same type, and heterotypic CICs, typically involving the engulfment of immune cells by tumor cells. The formation of CICs follows a dynamic, multi-step process encompassing cell recognition, adhesion, and internalization, which relies heavily on cytoskeletal remodeling. Key molecular participants in this process include *α*-catenin and *β*-catenin, actin filaments, myosin II, Rho GTPases, and Rho-associated coiled-coil-containing protein kinase (ROCK) (12, 16). Adhesion molecules such as E-cadherin (CDH1) and P-cadherin (CDH3) play critical roles in stabilizing intercellular junctions required for CIC formation (17, 18), while Ezrin, a cytoskeletal linker protein, has been shown to facilitate cell cannibalism during this process (19).

CICs have been documented in various malignancies, including lung, skin, and breast cancers, with growing evidence supporting their association with tumor progression, immune evasion, and therapeutic resistance (20–25). Notably, CICs have been observed in aggressive breast cancer subtypes such as HER2-positive and triple-negative tumors (26, 27). These structures may function as an adaptive mechanism under therapeutic stress, enabling tumor cells to eliminate damaged cells, evade immune attack, or access nutrients—thus supporting tumor survival and evolution.

In comparison with conventional prognostic markers like Ki-67, P53, and tumor-infiltrating lymphocytes (TILs), CICs offer unique insights into the tumor’s dynamic response to therapeutic stress (28). While Ki-67 serves as a marker of proliferative activity, and TILs reflect the immune response, CICs capture the real-time interactions between tumor cells (11), which are indicative of immune evasion, survival strategies, and resistance mechanisms. For instance, while Ki-67 may indicate cellular proliferation (21), it does not provide information about the tumor’s capacity to adapt to the hostile environment created by chemotherapy or immune surveillance. Similarly, while TILs offer valuable prognostic information about the tumor’s immune landscape (26), they do not specifically capture the ability of tumor cells to internalize one another and survive under chemotherapy-induced stress. CICs, therefore, complement these traditional markers by reflecting the tumor’s functional response to treatment (17), providing a potentially more dynamic and early indicator of therapy resistance.

A recent study reported a high prevalence of CICs in breast cancer, defining them as encapsulated cell structures that enable a distinct form of entotic cell death (29). These findings underscore the clinical relevance of CICs, especially their potential prognostic value in predicting both NACT response and patient outcomes in invasive breast cancer.

In this report, we present two cases of HER2-positive invasive ductal carcinoma with similar histopathological features, both treated with NACT in combination with trastuzumab. Despite receiving identical treatment regimens, the patients experienced markedly different clinical outcomes: one achieved pCR, while the other showed only stable disease (SD). Our retrospective analysis revealed contrasting changes in CIC prevalence between the two cases before and after NACT, suggesting that CIC dynamics may correlate with treatment efficacy. These findings highlight the potential of CICs as predictive biomarkers for NACT response and support further investigation into their clinical utility in optimizing therapeutic strategies for HER2-positive breast cancer.

## Case presentation

### Case 1

**Figure 1:**
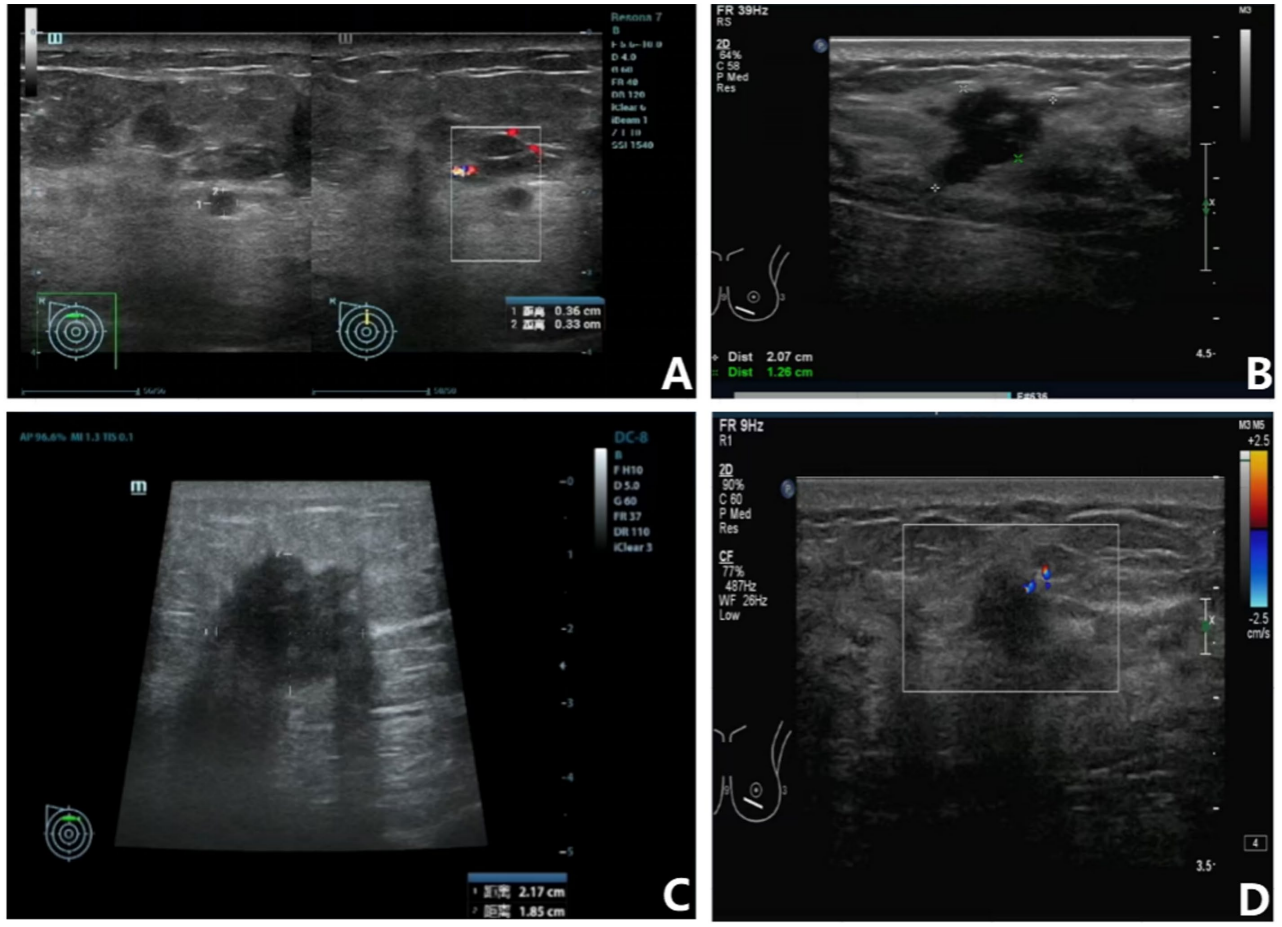
Ultrasound imaging of the breast tumors in the right and left breasts. **(A)** Pre-NACT ultrasound of the right breast showing a necrotic mass measuring 2.3 × 1.5 cm; **(B)** Post-NACT ultrasound of the right breast showing a slight increase in tumor size to 2.2 × 1.9 cm; **(C)** Pre-NACT ultrasound of the left breast revealing a mass measuring 2.1 × 1.3 cm; **(D)** Post-NACT ultrasound of the left breast showing a reduction in tumor size to 1.2 × 0.9 cm.

A 53-year-old woman arrived in July 2023 with a mass in her right breast. An ultrasonic examination using color Doppler imaging revealed a necrotic mass measuring 2.3 × 1.5 cm in size (Figure 1A; Table 1). A core needle biopsy of the breast mass and axillary lymph node verified the diagnosis of invasive ductal carcinoma with lymph node metastases, providing a classification of cT2N1M0 IIB (Table 2). The patient received neoadjuvant chemotherapy consisting of 4 biweekly cycles of dose-dense epirubicin (Pharmorubicin-RD) and cyclophosphamide (CTX), succeeded by 4 biweekly treatments of trastuzumab and docetaxel (TXT) (Table 2). Post-treatment ultrasound revealed a little enlargement of the tumor, now measuring 2.2 × 1.9 cm, and SD was diagnosed (stage cT2N1M0 IIB, Figure 1B; Table 1). Following this, a modified radical mastectomy of the right breast was performed, and the mass was sent for pathological examination. The final pathological stage was determined to be ypT1c ypN3c (Stage IIIC). After the surgery, the patient continued with adjuvant trastuzumab therapy.

**Table 1:** Clinicopathological characteristics and NACT treatment outcomes of two cases.

Characteristics	Case 1 (Pre-NACT)	Case 1 (Post-NACT)	Case 2 (Pre-NACT)	Case 2 (Post-NACT)
Tumor size (cm)	2.3 × 1.5	2.2 × 1.9	0.31 × 0.51	0.11 × 0.10
NACT Options	EC-TH	EC-TH	EC-TH	EC-TH
Curative effect	SD	SD	PR	PR
ER status	−	−	−	−
PR status	−	−	−	−
HER2 status	3+	3+	3+	3+
E-cadherin	+	+	+	+
P53 status	−	−	90%	70%
Ki-67 status	70%	70%	40%	10%
TNM stage	cT2N1M0 IIB	pT2N1M0 IIB	cT2N1M0 IIB	pT1N2M0 IIIA

**Table 2:** Nact option of two cases.

Case	Time	Neoadjuvant therapy	Treatment
1	2023.07.27–2023.10.13	EC*4	Pharmorubicin RD 160 mg + CTX 1.0 g ivdrip
	2023.10.14–2023.12.16	TH*4	TXT 150 mg + Herceptin 360 mg (First time 480 mg)
2	2024.01.22–2024.04.17	EC*4	Pharmorubicin RD 160 mg + CTX 1.0 g ivdrip
	2024.04.18–2024.07.09	TH*4	TXT 160 mg + Herceptin 420 mg (First time 560 mg)

### Case 2

A 53-year-old woman was diagnosed with breast cancer in January 2024. Ultrasound imaging revealed a 2.1 × 1.3 cm mass in the left breast (Figure 1C; Table 1). A core needle biopsy confirmed invasive ductal carcinoma with lymphatic spread to the left subaxillary lymph node, staging the cancer as cT2N1M0 IIB (Table 2). The patient received NACT comprising 4 cycles of Pharmorubicin-RD and CTX biweekly, succeeded by 4 cycles of trastuzumab and TXT biweekly, identical to the regimen in Case 1 (Table 2). Post-treatment ultrasound showed a reduction in tumor size to 1.2 × 0.9 cm, indicating a partial response (PR) based on imaging reevaluation (Figure 1D; Table 1). Following diagnostic confirmation of invasive ductal carcinoma, the patient underwent a modified radical mastectomy of the left breast. Pathologically validated infiltrative ductal cancer, with a final staging of pT1bN2aM0 IIIA.

## Pathological findings

### Case 1

**Figure 2:**
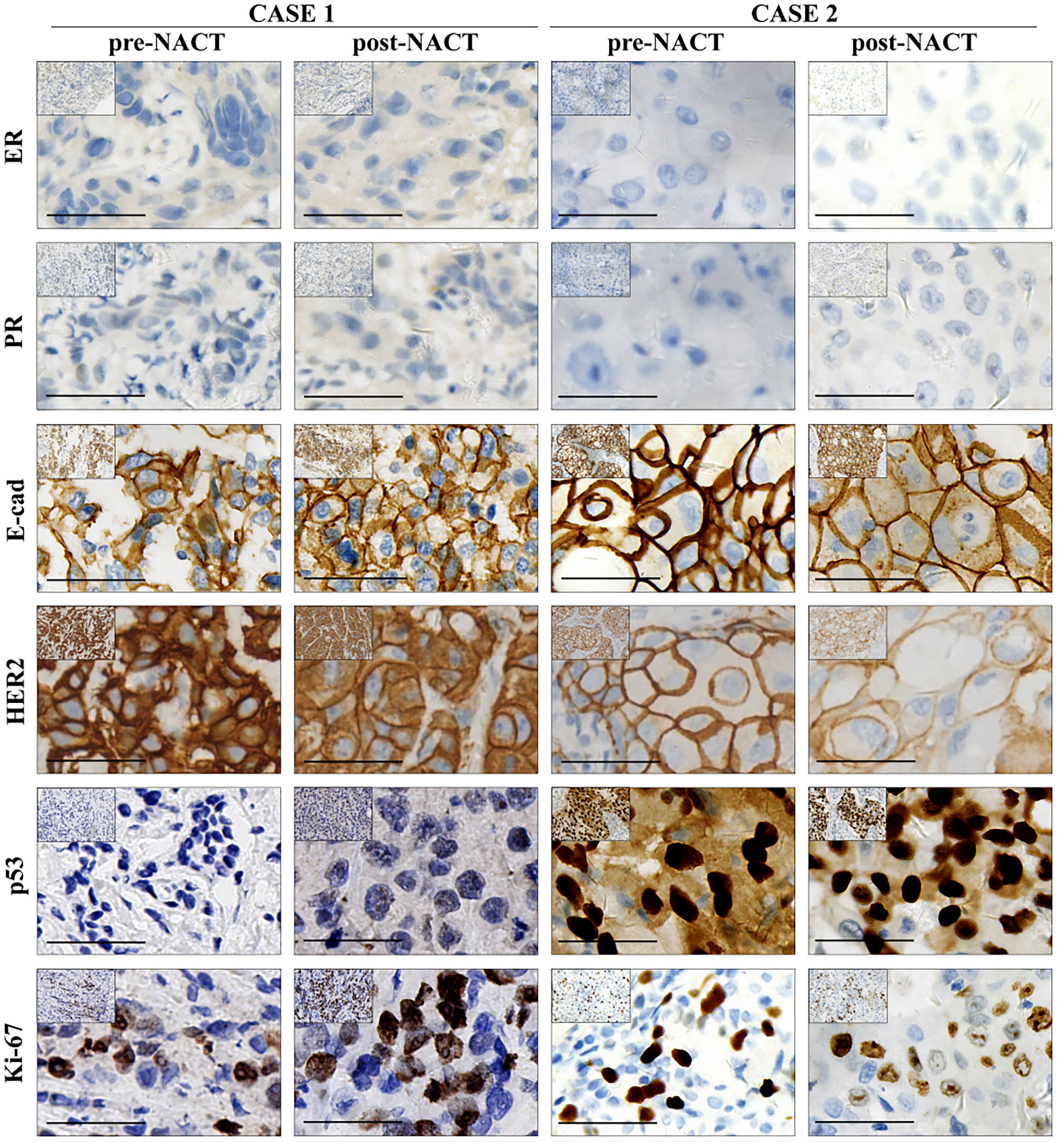
IHC analysis of case 1 and case 2 before and after NACT. The figure presents IHC staining results for estrogen receptor (ER), progesterone receptor (PR), E-cadherin (E-cad), HER2, p53, and Ki-67 in case 1 and case 2, both before and after NACT. Scale bar: 50 μm.

In the IHC analysis, the tumor was positive for E-cadherin (+), HER2 (+++), and exhibited a Ki-67 labeling index of 70%. It was negative for ER, PR, and P53 (Figure 2; Table 2). Additionally, no changes were observed in these pathological markers before or after NACT.

**Figure 3:**
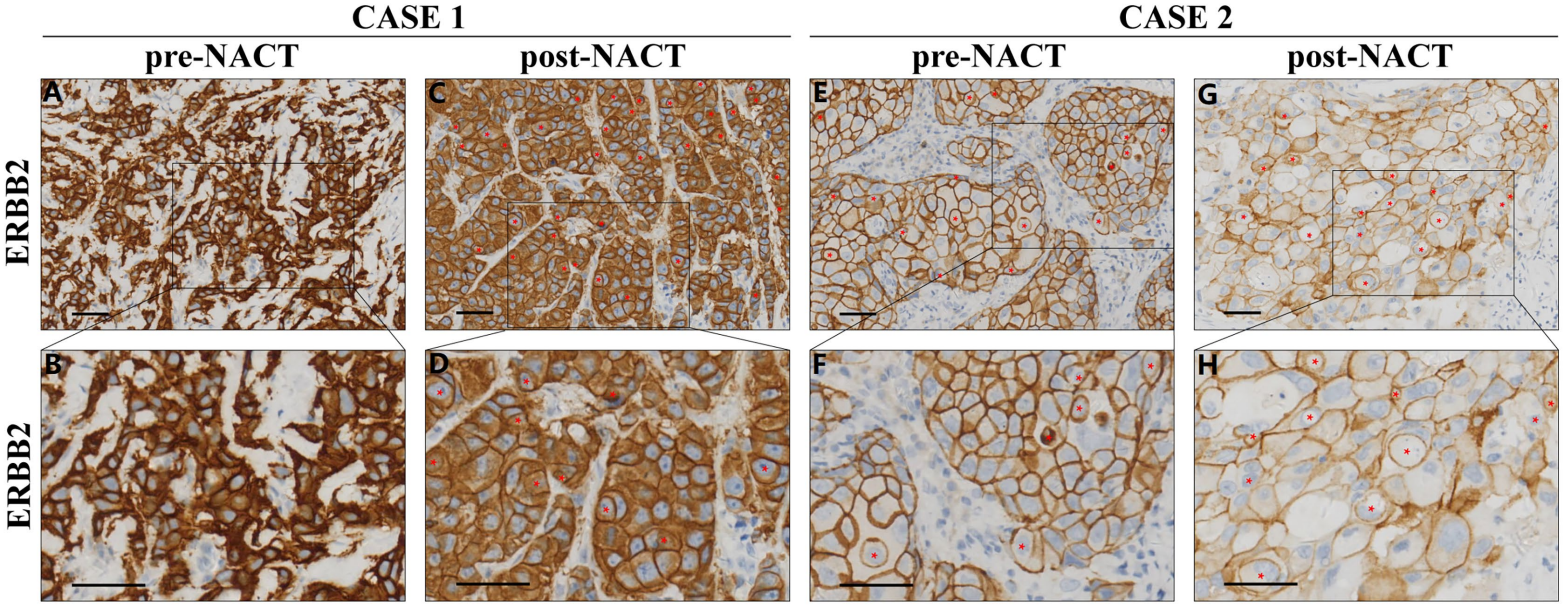
HER2 IHC microscopic findings in Case 1 and Case 2, pre- and post-NACT. Representative image for HER-2 staining. Inner cells of CIC structures are indicated with red asterisks. Scale bar: 100 μm. **(A,B)** Pre-NACT: classic invasive ductal carcinoma with dense lymphocytic infiltration, negative for CIC structures. **(C,D)** Post-NACT: increased CIC frequency (32 per 20 × field). **(E–H)** Tumor morphology post-NACT showing solid epithelial cords (asterisks), polygonal cells, and peripheral lumens, reduced CIC counts compared to pre-NACT.

**Figure 4:**
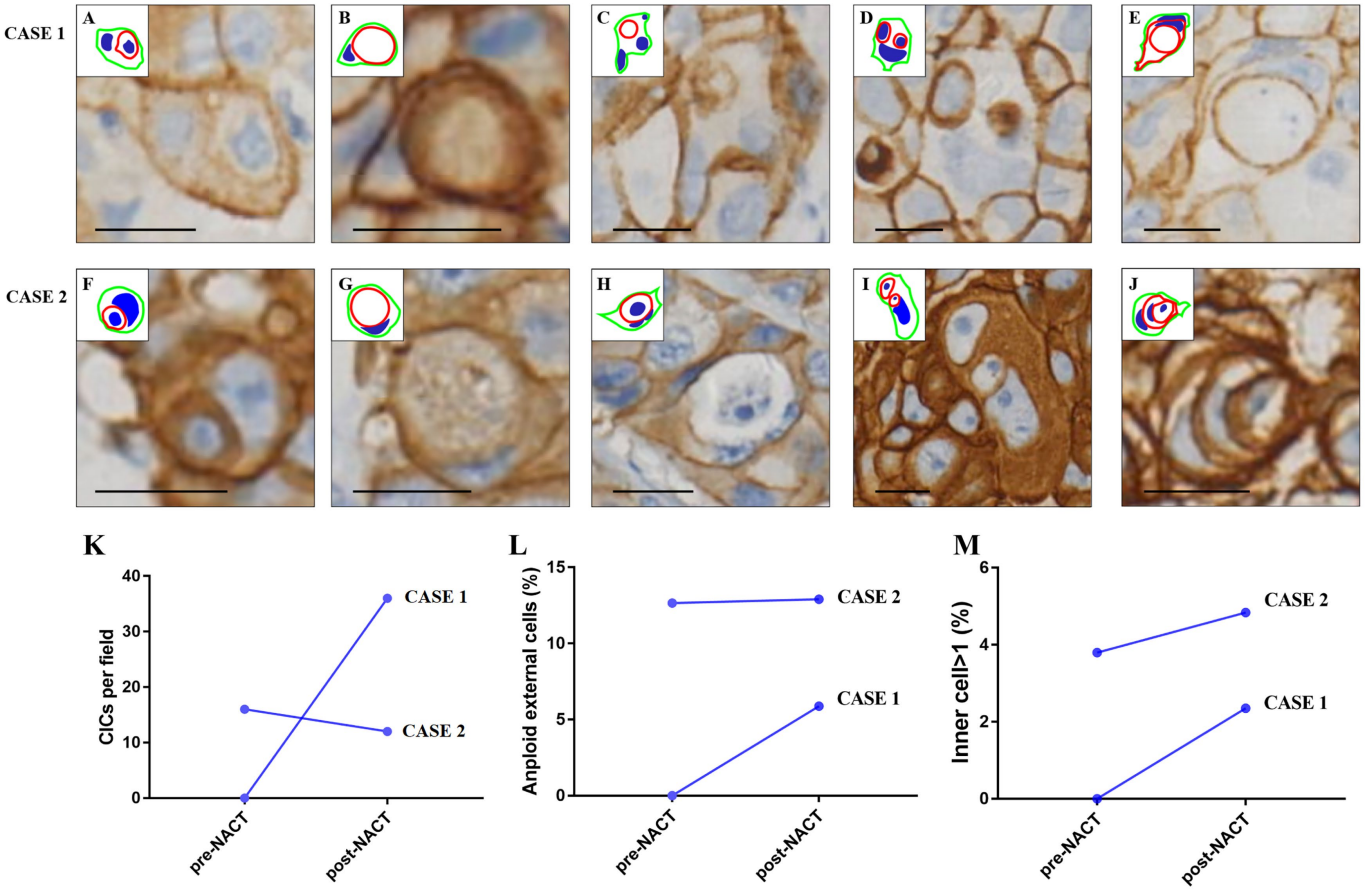
Cellular morphology and CIC characteristics in breast cancer in case 1 and case 2. Complex morphologies of CIC structures. Inserted pictures are schematic cartoons for the indicated CIC structures, respectively. Scale bar: 20 μm. **(A–C,F–H)** Majority of CICs are seen as one cell enclosed by another, with no significant internalization of more than one cell (20 × magnification); **(D,I)** CICs show structures with two or more cells enclosed in one another; **(E,J)** complex structures resulting from one cell within a cell that was inside of another cell. **(K)** Comparison of CICs in case 1 and case 2: The number of CICs post-NACT was higher in case 1 than in case 2; **(L)** multinucleated host cells of CICs: Multinucleated host cells of CICs are more frequent post-NACT in case 1 but show no significant difference between pre- and post-NACT in case 2; **(M)** presence of inner cells post-NACT: The percentage of inner cells (cells internalized within other cells) slightly increases post-NACT in both case 1 and case 2.

Microscopically, the lesion composed of classic invasive ductal carcinoma, surrounded by a dense of lymphocytes infiltration, and no CICs were found in the sample (Figures 3A,B). HER-2 staining labeling cell boundary depicts a number of unique structures morphologically resembling CIC structures. However, the average number of CICs was approximately 32 per 20 × microscopic field, much higher in the post-NACT than that in the pre-NACT (Figures 3C,D, 4K). The CICs were composed of variety of structure patterns, and most were one cell enclosure by another one (Figures 4A–C), and two or more cells into another one were also detected (Figure 4D). In certain instances, intricate structures arise from a cell contained within another cell, as illustrated in Figure 4E. We observed that several internalizing cells seemed to exist within a huge vacuole lacking an intact nucleus, suggesting they may be undergoing entotic cell death (Figures 4B,C,E). Of note, multinucleated host cells of CICs were increased in post-NACT, and multinucleation frequencies were much higher than that in pre-NACT (Figure 4L).

### Case 2

Immunohistochemistry verified that the lesion exhibited positivity for E-cadherin (+) and HER2 (+++), consistent with the prior example. Ki-67 and P53 exhibited positivity in 40 and 90% of the cells, respectively, but ER and PR were entirely negative (Figure 2; Table 2). It is worth to note that the pathological index of Ki-67 and P53 decreased from 40 and 90% to 10 and 70%, respectively.

Microscopic examination revealed that the lesion consisted of an epithelial neoformation with lobulated contours, made up of solid epithelial cords containing polygonal cells and small lumens at the periphery. The cells exhibited diffuse growth, and the tumor was associated with areas of necrosis. Compared to the pre-NACT sample, the number of CICs was reduced in the post-NACT sample (Figures 3E–H, 4K). Both typical and more complex CICs were observed in both pre- and post-NACT samples (Figures 4F–J). No substantial difference was observed in the quantity of multinucleated host cells within the CICs between the pre- and post-NACT samples (Figure 4L). Similar to case 1, the percentage of CICs containing one or more internalized cells was slightly higher after NACT (Figure 4M).

## Discussion

Breast cancer is the most frequently diagnosed malignancy in women and the second primary cause of death from cancer globally (30). According to the molecular markers, including ER, PR and HER2 (31), breast cancer was divided into three primary subtypes: hormone receptor (HR)-positive, HER-2-positive and triple-negative breast cancer (TNBC) (32). NACT has been a standard clinical practice to diminish tumor size and improve the likelihood of breast conservation (7). pCR has been established as the primary goal for neoadjuvant trials, correlating with long-term survival outcomes (33). Improving patient survival through various treatment modalities remains a key focus. Ignatiadis et al. demonstrated that the presence of high TILs during NACT correlates with an increased rate of pCR and may serve as a predictor for pCR in patients with HER2-positive breast cancer (34). Numerous studies have established that circulating tumor DNA (ctDNA) serves as a predictive marker for pCR, indicating that ctDNA following NACT correlates with the presence of residual disease (34, 35). An effective biomarker should predict both prognosis and therapeutic response (31).

CICs as an unique structure, characterized by cells inside the cytoplasm of another cell, has been reported in various tumor types including breast cancer (28), hepacellular carcinoma (HCC) (36), pancreatic ductal adenocarcinoma (PDAC) (37). CICs have demonstrated prognostic usefulness for prognosis and treatment response based on retrospective investigations (38). Recent evidence suggests that CIC structures may not only serve as morphological features of aggressive tumor phenotypes but also reflect functional responses to therapeutic stress (39, 40). In our report, the patient who achieved pCR exhibited a marked reduction in CICs following NACT, whereas the patient with SD showed a persistent or increased presence of CICs. This contrast implies a potential correlation between dynamic CIC changes and treatment efficacy. One plausible explanation is that chemotherapy may disrupt the cytoskeletal and adhesive machinery necessary for CIC formation in sensitive tumors (39, 41, 42). In contrast, resistant tumors may retain or even enhance their CIC-forming capabilities to adapt and survive under cytotoxic stress (43). Functionally, CICs may promote immune evasion, eliminate damaged or apoptotic cells, and provide metabolic support, ultimately contributing to tumor cell survival and clonal selection (44).

The formation of CICs in HER2-positive breast cancer is influenced by several key molecular mechanisms, particularly involving E-cadherin, ROCK, and entosis. E-cadherin, a crucial adhesion molecule, plays a pivotal role in maintaining cell–cell adhesion and is essential for CIC formation. HER2 signaling has been shown to regulate the expression and function of E-cadherin, facilitating the interaction between tumor cells and promoting CIC formation. This dynamic process not only enhances tumor cell survival but also contributes to immune evasion and therapy resistance (17). Similarly, ROCK, a regulator of cytoskeletal remodeling, is involved in the internalization of cells during CIC formation. In HER2-positive tumors, ROCK activation can enhance the ability of tumor cells to internalize one another, providing an adaptive advantage in response to chemotherapy-induced stress (12). Additionally, entosis, a form of non-apoptotic cell death, allows tumor cells to internalize damaged cells, potentially promoting tumor survival under therapeutic pressure. These pathways—E-cadherin, ROCK, and entosis—interact with HER2 signaling to facilitate CIC formation, contributing to therapy resistance and highlighting the potential of targeting these pathways to improve treatment outcomes in HER2-positive breast cancer (28).

Compared with conventional biomarkers such as Ki-67, TILs, and P53 mutation status, CICs represent a fundamentally different class of biological indicators. While Ki-67 reflects the proliferative activity of tumor cells and TILs reflect host immune response (45, 46), CICs capture real-time, cell–cell interaction phenomena that may signify tumor cell adaptability under stress (43). Unlike static markers that offer a snapshot of genetic or protein expression levels, CICs are dynamic, functional structures that may serve as early indicators of treatment resistance or cellular survival strategies during therapy (27, 47). One of the key advantages of CICs lies in their integrative representation of multiple tumor-related mechanisms, including immune escape, metabolic competition, and the clearance of damaged or dying cells (20–25). This multidimensional view of tumor cell behavior may enhance our understanding of treatment resistance and disease progression, especially in aggressive subtypes. Furthermore, CICs have been observed across a range of malignancies, suggesting their potential as broadly applicable indicators.

Despite these strengths, several limitations remain. Unlike established markers such as Ki-67 or P53, which are supported by standardized scoring systems and widely used in clinical diagnostics, the identification and quantification of CICs lack consistent methodological guidelines. Their detection currently relies on histopathological morphology, often through hematoxylin and eosin (H&E) staining, which introduces subjectivity and potential variability among observers. Additionally, the absence of validated molecular markers specific to CICs limits their application in high-throughput or automated platforms. The reproducibility and scalability of CIC assessment remain challenges that must be addressed before clinical translation can be achieved. The variability in CICs, specifically the frequency of CICs, was observed between patients. One patient showed a significant reduction in CICs after treatment, while the other exhibited persistent or increased CIC frequency, suggesting that the tumor’s response to therapy may be associated with the dynamic changes in CIC formation. This variability in CICs underscores their potential role as a marker for treatment efficacy and resistance (28).

This study has several limitations that should be acknowledged. First, the sample size is very small, consisting of only two clinical cases. This limits the generalizability of our findings and precludes robust statistical analysis. The observed differences in clinical response, stable disease in one patient and partial response in the other, represent a relatively narrow spectrum of outcomes, making it difficult to draw definitive conclusions regarding the predictive value of CICs. Second, the retrospective nature of the study may introduce selection bias and limits our ability to establish causal relationships. Third, the follow-up period was relatively short, which precludes long-term evaluation of treatment outcomes such as recurrence or overall survival. Additionally, CIC identification in this study was based on conventional histopathological assessment, which may be subject to observer variability due to the absence of standardized criteria or automated quantification tools. Despite these limitations, our findings highlight the potential role of CIC structures as functional biomarkers in HER2-positive breast cancer. Future studies should focus on validating CIC dynamics in larger, prospective cohorts and across different breast cancer subtypes. The development of standardized evaluation guidelines and digital image analysis platforms based on artificial intelligence could improve the accuracy and reproducibility of CIC quantification. Furthermore, the identification of CIC-specific molecular markers and gene expression signatures may provide deeper mechanistic insights and facilitate the integration of CIC assessment into clinical workflows. Ultimately, a more comprehensive understanding of CIC biology and its interaction with therapy could lead to novel strategies for personalized cancer treatment.

In conclusion, we describe and compare two instances of HER2-positive breast cancer pre- and post-NACT characterized by CICs, with clinic parameters such as ER, PR, E-cadherin, P53 and Ki-67. These cases showed that the case with obviously increased of CICs had poor prognosis, while the other case with no significant change of CICs had PR effect. It may be a clue for further investigation on CICs could serve as prognostic and perhaps predictive markers in response to NACT in HER2-positive breast cancer.

## Methods

### Tissue preparation and immunohistochemistry

Tumor tissues from Case 1 and Case 2, collected before and after NACT, were fixed in 10% neutral-buffered formalin, embedded in paraffin, and sectioned at 5 μm. Sections were deparaffinized, rehydrated, and subjected to citrate buffer antigen retrieval (pH 6.0) for 15 min. Endogenous peroxidase was blocked with 3% H₂O₂, followed by 5% bovine serum albumin (BSA) for 1 h at room temperature. Primary antibodies against ER, PR, E-cadherin, HER2, p53, and Ki-67 were applied overnight at 4 °C, followed by horseradish peroxidase (HRP)-conjugated secondary antibodies for 1 h. Staining was visualized with 3,3′-Diaminobenzidine (DAB) and counterstained with hematoxylin.

### CIC identification and quantification

CICs were defined as one or more tumor cells fully or partially enclosed by a continuous host cell membrane, with an intact nucleus in the internalized cell, and simple planar overlaps were excluded. Paraffin sections were cut at 4–5 μm, which is much thinner than the average tumor cell (~15–20 μm), allowing the internalized cells to be clearly visualized and reducing the likelihood of misinterpreting overlapping cells as CICs. Schematic diagrams were manually overlaid on representative IHC images using PowerPoint (Microsoft), following the approach described by Ruan et al. (2019) (29). In the diagrams, outer membranes were marked in red, internalized cells in green, and nuclei in blue. Two investigators independently annotated CICs, and discrepancies were resolved by consensus to ensure reproducibility.

CIC counts, percentages of abnormal outer nuclei, and internalized cells were quantified from five randomly selected fields under 20 × objective magnification (~200 × total) for each specimen. Line plots and descriptive statistics were generated using general graphing software.

## Data Availability

The original contributions presented in the study are included in the article/Supplementary material, further inquiries can be directed to the corresponding authors.

## Glossary

BSA: Bovine Serum Albumin
CDH1: E-cadherin
CDH3: P-cadherin
CIC: Cell-in-cell
CICs: Cell-in-cell structures
ctDNA: Circulating tumor DNA
CTX: Cyclophosphamide
DAB: 3,3′-Diaminobenzidine
ER: Estrogen receptors
HCC: Hepacellular carcinoma
H&E: Hematoxylin and eosin
HER2, ERBB2: Human Epidermal Growth Factor Receptor 2
HR: Hormone receptor
HRP: horseradish peroxidase
IHC: Immunohistochemical
NACT: Neoadjuvant chemotherapy
p53: Tumor protein p53
pCR: Pathological complete response
PDAC: Pancreatic ductal adenocarcinoma
Pharmorubicin-RD: Dose-dense epirubicin
PI3K-AKT: Phosphatidylinositol 3-kinase–AKT
PR: Progesterone receptors
PR: Partial response
ROCK: Rho-associated coiled-coil-containing protein kinase
SD: Stable disease
TILs: Tumor-infiltrating lymphocytes
TNBC: Triple-negative breast cancer
TXT: Docetaxel
